# Dynamics of the natural transmission of bovine spongiform encephalopathy within an intensively managed sheep flock

**DOI:** 10.1186/s13567-015-0269-x

**Published:** 2015-10-28

**Authors:** Martin Jeffrey, Janey P. Witz, Stuart Martin, Steve A. C. Hawkins, Sue J. Bellworthy, Glenda E. Dexter, Lisa Thurston, Lorenzo González

**Affiliations:** Animal and Plant Health Agency (APHA-Lasswade), Pentlands Science Park, Bush Loan, Penicuik, Midlothian, EH26 0PZ UK; APHA-Weybridge, New Haw, Addlestone, Surrey, KT15 3NB UK

## Abstract

Sheep are susceptible to the bovine spongiform encephalopathy (BSE) agent and in the UK they may have been exposed to BSE via contaminated meat and bone meal. An experimental sheep flock was established to determine whether ovine BSE could be naturally transmitted under conditions of intensive husbandry. The flock consisted of 113 sheep of different breeds and susceptible *PRNP* genotypes orally dosed with BSE, 159 sheep subsequently born to them and 125 unchallenged sentinel controls. BSE was confirmed in 104 (92%) orally dosed sheep and natural transmission was recorded for 14 of 79 (18%) lambs born to BSE infected dams, with rates varying according to *PRNP* genotype. The likelihood of natural BSE transmission was linked to stage of incubation period of the dam: the attack rate for lambs born within 100 days of the death of BSE infected dams was significantly higher (9/22, 41%) than for the rest (5/57, 9%). Within the group of ewes lambing close to death, those rearing infected progeny (*n* = 8, for 9/12 infected lambs) showed a significantly greater involvement of lymphoid tissues than those rearing non-infected offspring (*n* = 8, for 0/10 infected lambs). Horizontal transmission to the progeny of non-infected mothers was recorded only once (1/205, 0.5%). This low rate of lateral transmission was attributed, at least partly, to an almost complete absence of infected placentas. We conclude that, although BSE can be naturally transmitted through dam-lamb close contact, the infection in this study flock would not have persisted due to low-efficiency maternal and lateral transmissions.

## Introduction

The transmissible spongiform encephalopathies (TSEs) or prion diseases comprise a group of fatal neurological disorders affecting man, domestic ruminants, farmed and free-living cervid species, domestic and captive felids and, rarely, farmed mustellids. The infectious agent of TSEs is poorly characterised and diagnosis of prion diseases is generally achieved by detecting disease-associated forms of the prion protein (PrP^d^) using immunohistochemistry or immunoassay, or partially protease resistant forms of prion protein (PrP^res^) by molecular methods.

The bovine spongiform encephalopathy (BSE) epidemic of cattle in the UK and Europe was initiated and largely maintained by the inclusion of BSE contaminated meat and bone meal in concentrate feedstuffs [1, 2]. It is likely that at least some sheep and goats in the UK received concentrate feedstuffs that were at significant risk of containing the BSE agent. Many experimental studies have confirmed the susceptibility of sheep for the BSE agent and, moreover, there are suggestions that the ovine adapted BSE agent may be more virulent than the cattle agent [3]. However, no natural cases of BSE in sheep have yet been confirmed, though cases of caprine BSE have been identified in Europe [4, 5]. Any infection of sheep that may have occurred in the 1980s, when cattle BSE was first identified, would potentially have undergone many serial passages over generations with potential for further adaptation and phenotypic changes in the nature of the disease.

Previous studies have reported that sheep are susceptible to BSE infection by several transmission routes including oral challenge [6–10], which might be expected to most closely mimic original exposure to the BSE agent. However, no studies have been carried out to study the potential for natural BSE transmission following initial exposure to contaminated feedstuffs. In contrast, scrapie is a naturally transmissible, contagious infection and a number of studies have shown several possible exposure sources deriving from infected dams. Although a large study of pre-implantation embryos found no evidence for trans-ovarial infectivity [11], other studies suggested that some foetuses may become infected in utero at late stages of gestation [12–14]. Infectivity [15] and PrP^d^ [16, 17] is found in placentas of scrapie infected sheep allowing both for possible in utero infection via amniotic fluid ingestion by the foetus and for post natal transmission following contamination of the environment. In separate experiments, milk obtained from scrapie infected sheep and goats was used to experimentally infect naïve new-born lambs of susceptible prion protein (*PRNP*) genotypes with high efficiency [18, 19]. Other studies suggest that horizontal transmission to progeny of uninfected dams may be efficient under conditions of high infection pressure but less efficient when environmental contamination is less significant [20].

We have previously reported preliminary evidence of natural BSE transmission from dam to offspring [21]. This report describes the outcome of the complete, large-scale study from which those preliminary observations were reported. The aim of the study was to determine the efficiency of natural transmission of BSE in sheep, including those of maternal and horizontal transmission, the factors that could affect such efficiencies and the disease phenotype of naturally transmitted sheep BSE.

## Materials and methods

### *PRNP* genotyping

The *PRNP* genotypes of sheep to be orally dosed, to be introduced as sentinel controls or to be used for breeding purposes (see below) were determined prior to undertaking the corresponding procedures (dosing, mixing or mating). Such genotyping was confined to codons 136, 154 and 171 and sheep of three genotypes were selected: ARQ/ARQ, AHQ/AHQ and VRQ/VRQ (A, alanine or V, valine at codon 136; R, arginine or H, histidine at codon 154; Q, glutamine at codon 171). Since all animals included in this study were homozygotes at those three codons, a single allele denomination will be used throughout this report. During the course of this study, data emerged suggesting that New Zealand-derived Suffolk sheep with the ARQ allele might show an additional polymorphism at codon 112 (T, threonine instead of M, methionine), with influence on susceptibility to oral BSE [22]. In view of this, all ARQ sheep within the study (Romney and Suffolk, either orally dosed, their progeny or sentinel controls, see below) and a selection of Cheviot sheep (20 VRQ and 25 AHQ) were subjected to full *PRNPORF* genotyping by methods described previously [23].

### BSE homogenates used for oral challenges

Three different cattle BSE homogenates were used for oral challenge: (1) BBP1, with a titre of 10^4.07^ RIII mouse ic/ip LD_50_/g^−1^, (2) BBP6, not titrated in mice and (3) BBP8, a 50% dilution of an inoculum with a titre of RIII mouse 10^3.84^ ic/ip LD_50_/g^−1^. In previous experiments [10, González et al., unpublished observations], the three cattle BSE inocula caused disease in ARQ sheep with similar attack rates (ARs) and survival times [STs: 589 ± 15 days (mean ± standard deviation, SD) for BBP1, 619 ± 24 days for BBP6 and 530 ± 14 days for BBP8] that did not clearly parallel the infectivity expected from mouse titres.

### Experimental animals

**Figure 1:**
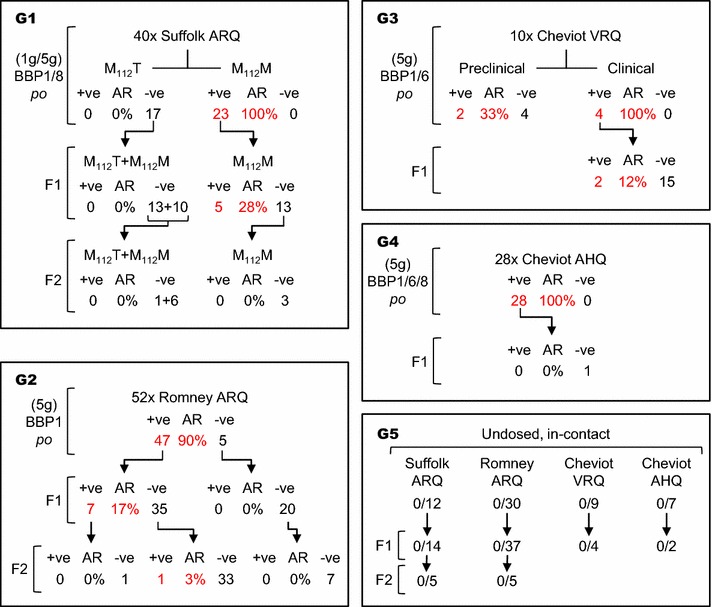
**Overall layout of the study and its main results.** The study comprised four groups (G1-G4) of sheep orally challenged with BSE and their progeny and one group (G5) of sentinel controls and their offspring. AR, attack rate, as percentage. Combined attack rates are: A) for orally dosed susceptible sheep (M_112_T Suffolk sheep excluded) including clinically affected animals and preclinical culls, 104/113 = 92.0%; B) for the susceptible F1 and F2 progeny of infected dams, 14/79 (17.7%); 5) for susceptible sheep exposed only to lateral transmission, 1/205 = 0.5%. For other details see Table 1.

To establish the flock, sheep of selected breeds and *PRNP* genotypes were obtained from a New Zealand-derived, classical scrapie-free flock (ARSU, APHA, UK) and orally dosed with the different cattle BSE inocula listed above (Figure 1). *PRNP ORF* genotyping done well after experimental challenges were completed detected the M_112_T polymorphism in 48 ARQ Suffolk sheep (17 orally dosed and 31 progeny); since none of these sheep (Figure 1) or the 10 M_112_T sentinel controls showed evidence of BSE, those 58 sheep have not been included in this report. Additional polymorphisms were not detected in ARQ Romney sheep or in VRQ and AHQ Cheviot animals.

A total of 113 sheep were orally dosed. Six Suffolk sheep were given 1 g of inoculum at 6–10 days of age and the remaining 107 were dosed with 5 g of inoculum at different ages (Table 1): ARQ Suffolk sheep at either ~4–5 (*n* = 13) or ~6–8.5 months (*n* = 4); ARQ Romney sheep at ~8 (*n* = 24) or ~11–12 months (*n* = 28); VRQ Cheviot sheep at ~2 (*n* = 6) or ~4 months (*n* = 4); AHQ Cheviot sheep at ~2.5–4.5 (*n* = 19) or ~8–10 months (*n* = 9). Oral dosing of these sheep took place during the first 8 years of the study: 2001, 28; 2002, 11; 2003, 5; 2004, 28; 2006, 15; 2007, 5; 2008, 21.

**Table 1:** Details of the study design and results (attack rates and survival times).

Type of exposure	Breed and genotype^a^	Age at exposure^b^	BSE infected			Survivors	
			n	AR	ST	n	ST
Oral challenge	Suffolk ARQ 1 g	*9* *±* *1*	*6* ^c^	*100*	*575* *±* *87 (525–729)*	*0*	
	Suffolk ARQ 5 g	112–147	13	100	712 ± 119 (575–1034)	0	
		190–255	4	100	712 ± 106 (570–800)	0	
		*145* *±* *41*	*17*	*100*	*712* *±* *113*	*0*	
	Romney ARQ 5 g	230–253	21	87.5	830 ± 93 (665–1058)	3	2380 (2347–2397)
		342–363	26	92.9	822 ± 149 (672–1365)	2	1580 (1562–1597)
		*304* *±* *58*	*47*	*90.4*	*825* *±* *126*	*5*	
	Cheviot VRQ 5 g	54–58	2^d^	33.3	966–967	4^d^	954 (915–967)
		*126* *±* *0*	*4*	*100*	*1772* *±* *206 (1558–2026)*	*0*	
	Cheviot AHQ 5 g	76–138	19	100	601 ± 54 (498–665)	0	
		229–294	9	100	597 ± 57 (524–686)	0	
		*145* *±* *80*	*28*	*100*	*600* *±* *54*	*0*	
*Total oral dosing*			*104*	*92.0*		*9*	
F1/infected dam	Suffolk ARQ	From birth	5	27.8	787 ± 101 (654–893)	13	1096 (535–2647)
	Romney ARQ	“	7	16.7	870 ± 154 (673–1084)	35	2020 (693–2674)
	Cheviot VRQ	“	2	11.8	1804, 1883^e^	15	1913 (1454–2247)
	Cheviot AHQ	“	0	0		1	1276
F2/infected F1	Romney ARQ	“	0	0		1	1220
*Total maternal*			*14*	*17.7*		*65*	
F1/not infected dam	Suffolk ARQ	From birth	0	0		10	999 (315–2662)
	Romney ARQ	“	0	0		20	1116 (309–2621)
F2/not infected F1	Suffolk ARQ	“	0	0		9	997 (312–1580)
	Romney ARQ	“	1	2.4	392^e^	40	1057 (309–2242)
Sentinels	Suffolk ARQ	From mixing	0	0		12	1614 (1277–1736)
	Romney ARQ	“	0	0		30	2177 (1058–2959)
	Cheviot VRQ	“	0	0		9	1526 (1203–1653)
	Cheviot AHQ	“	0	0		7	1491 (695–1764)
F1–F2/sentinels	Suffolk ARQ	From birth	0	0		19	1034 (293–2243)
	Romney ARQ	“	0	0		42	1559 (344–2621)
	Cheviot VRQ	“	0	0		4	1018 (728–1840)
	Cheviot AHQ	“	0	0		2	403 (298–507)
*Total horizontal*			*1*	*0.5*		*204*	

n, number of sheep in each subgroup and group; AR, attack rate as percentage; ST, survival time in days as mean ± SD (range) for BSE infected sheep (actual days for the two F1 VRQ Cheviot sheep) and as mean (range) for survivors

^a^Only M_112_M ARQ Suffolk sheep included

^b^Age at exposure in days as range for the different subgroups and as mean ± SD for the breed/genotype/dose groups (rows in italics; in the case of orally dosed Cheviot VRQ, only those left to develop clinical disease are highlighted)

^c^Includes one BSE +ve animal culled at 371 dpi (not considered in ST values)

^d^These six sheep were culled at preclinical stage

^e^These two sheep were culled at the stated ages in the absence of clinical disease and found to be PrP^d^ positive

Between one and 6 months following the initial oral challenges in 2001, an additional 58 non-dosed sheep (Figure 1) of the same breeds, genotypes and flock of origin were mixed with the dosed animals. Conventional intensive husbandry practices were followed as far as possible whilst avoiding possible sources of iatrogenic transmission. All animals were kept in a single group housed in purpose built accommodation for the duration of the study (until 2013) without access to open pasture; they were offered forage ad libitum supplemented with an appropriate quantity of concentrate. Bedding was wood shavings for the life of the study and this was replenished as necessary to maintain dry bedding, although complete removal of the bedding was required regularly. Both experimentally and naturally exposed ewes were bred from 18 months of age by natural mating using breed and genotype matched sires from the same scrapie-free flock and all sheep were given unrestricted access to lambing areas to maximise opportunity for spread of infection around lambing. Over successive years and lambing seasons, 108 susceptible first generation (F1) lambs were born to BSE dosed dams (78 to dams that went on to develop BSE and 30 to dams that were dosed but did not develop BSE) and another 51 were the progeny of the F1 generation (F2, 1 from a F1 dam that developed BSE and 50 from non-infected F1 dams). Similarly, 57 sheep were the F1 and 10 the F2 of the 58 undosed sentinel controls. All these sheep were kept within the flock as explained below.

In summary, this report includes a total of 397 sheep that were (1) of susceptible *PRNP* genotype, (2) exposed to BSE through either experimental challenge, maternal contact or horizontal transmission and (3) resident in the flock for sufficient time to have been confidently detected as infected after exposure. It does not include sheep of resistant genotypes or those that, although susceptible, died from intercurrent disease or were culled (for welfare reasons or to reduce stock density) at ages too young to make a BSE-negative diagnosis reliable. This age factor was breed and genotype dependent and worked out on the basis of previous experience on sheep BSE after oral infection [10, González et al., unpublished observations] and of the STs of sheep that succumbed to BSE during the present experiment (Table 1).

Sheep were monitored for clinical signs of neurological disease and humanely killed when BSE-compatible signs reached a predefined end-point, or culled because of old age, or at the termination of the study. Most of the flock was depopulated in 2010, by which time all orally dosed sheep had either succumbed to BSE, had been culled due to old age, or killed with evidence of preclinical infection. Similarly, all sentinel controls (dams, F1 and F2) were culled by the same time in the absence of clinical disease. By 2010, most of the F1 and F2 progenies of BSE dosed sheep had also been killed for the above reasons or with clinical BSE and only some sheep born to BSE confirmed dams remained in the flock until 2013. Details on the STs of the different groups are given in Table 1.

In addition to clinical examination and to further assist in monitoring BSE infection within the flock, some sheep were subjected to immunohistochemical (IHC) examination of lymphoid tissue biopsies, either from palatine tonsil [16] or recto-anal mucosal associated lymphoid tissue (RAMALT) [24].

All procedures were approved under the Animal (Scientific Procedures) Act 1986 and by the Veterinary Laboratories Agency (currently APHA) ethics committee and carried out under Home Office Project Licences numbers PPL70/4495, PPL70/5781 and PPL70/6795.

### Post-mortem and laboratory testing

All sheep that died or were culled were subject to necropsy and a range of tissue samples were taken including brain and a selection of lymphoreticular system (LRS) tissues. Additionally, some placentas were recovered after lambing and available for laboratory examinations. From each sheep in the flock a minimum set of samples, including brain at the obex and cerebellum, retropharyngeal lymph node and distal ileum was examined; sheep that were positive for PrP^d^ in any of those tissues were examined more widely, including an additional set of five standard brain areas, palatine tonsil, mesenteric and prescapular lymph nodes and spleen. All tissue samples were fixed in formaldehyde and processed for standard histology and immunohistochemistry (IHC).

The presence of PrP^d^ was confirmed in brain and other tissues by IHC methods previously described [25]. In addition, brain and LRS tissues from all confirmed BSE cases were examined by discriminatory IHC methods using P4 monoclonal antibody (R-Biopharm, Darmstadt, Germany) recognising the ovine PrP amino acid sequence 93–99 (WGQGGSH [26]) and R145 monoclonal antibody (APHA, Weybridge, UK), which recognises the amino acid sequence RESQA at positions 222–226 of ovine PrP [5]. Brains and LRS tissues were scored for magnitude and types of PrP^d^ accumulation using methods and protocols previously reported [25, 27].

### Statistical analyses

Attack rates between the different experimental groups were compared by means of Fisher’s exact tests, while differences between mean STs of the different groups and average PrP^d^ values in brain and LRS tissues were assessed using Student’s two sample unpaired *t* test. In both analyses, *P* < 0.05 values were considered statistically significant.

## Results

### Sheep orally challenged with cattle BSE brain homogenates

Attack rates were 100% for M_112_M ARQ Suffolk sheep (23/23), for AHQ Cheviot sheep (28/28) and for VRQ Cheviot sheep left to develop clinical disease (4/4). These figures were higher, though not significantly different (*P* > 0.1 in all comparisons), than the 90.4% AR (47/52) observed for ARQ Romney sheep. ARs were identical regardless of the inoculum (BBP1, BBP6 or BBP8) and the dose (1 or 5 g for Suffolk sheep) used. Six VRQ Cheviot sheep were culled at preclinical stage (at approximately half of the ST of those left to develop clinical disease; Table 1) and BSE could only be confirmed in two of them (33%; Figure 1; Table 1).

**Figure 2:**
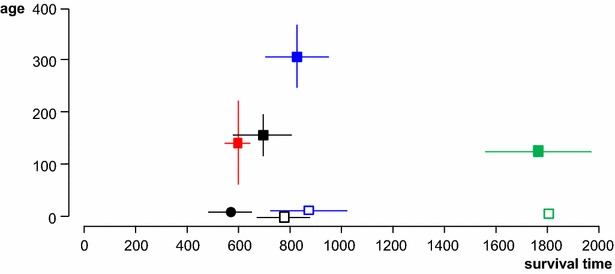
**Survival times in relation to ages at infection**. Survival time (X axis in days, mean ± SD) of orally dosed sheep (solid symbols; squares, 5 g dose and circle, 1 g dose) and naturally exposed progeny (open symbols) in relation to their age at exposure (Y axis in days, mean ± SD; for naturally exposed progeny, the age has been arbitrarily defined as around birth). Black, ARQ Suffolk sheep; blue, ARQ Romney sheep, green, VRQ Cheviot sheep; red, AHQ Cheviot sheep. Note that (1) the survival times of orally dosed AHQ Cheviot sheep are similar (low standard deviation) despite noticeable differences in age at exposure (see Table 1 for details) and (2) the survival times of orally dosed sheep are similar to those of naturally exposed progeny of the same breed and genotype.

Survival times of orally dosed sheep are shown in Table 1 and Figure 2. Of the six M_112_M ARQ Suffolk sheep dosed with 1 g of cattle BSE, one was culled at 371 days post-infection (dpi) and showed PrP^d^ accumulation in brain and LRS tissues at necropsy. The other five succumbed to clinical BSE with STs of 575 ± 87 dpi (mean ± standard deviation). Amongst sheep dosed with 5 g of inoculum, STs were shortest for AHQ Cheviot sheep (600 ± 54 dpi) and most protracted for VRQ Cheviot sheep (1772 ± 206 dpi), with intermediate values for M_112_M ARQ Suffolk (712 ± 113 dpi) and ARQ Romney sheep (825 ± 126 dpi). Differences in ST were significant between each of these four groups (Figure 2), including between Suffolk and Romney sheep of the same ARQ genotype. As with the ARs, no differences in ST were observed that could be attributed to the inocula. Moreover, within each of the three groups of ARQ Suffolk and Romney and AHQ Cheviot sheep dosed with 5 g of cattle BSE, no differences in ST were observed between subgroups that differed in their age at challenge (statistical analyses not shown but can be inferred from data in Table 1).

### Sheep of susceptible *PRNP* genotypes exposed to natural horizontal transmission only

These animals included the sentinel controls (*n* = 58) and their progeny (*n* = 67), the F1 (*n* = 30) of orally dosed ewes that did not develop BSE and the F2 (*n* = 49) of non-infected F1. Of a total of 205 sheep, all of which were of susceptible *PRNP* genotype and either in contact with infected sheep or exposed to potentially contaminated environment for periods running into years (Table 1), only one Romney sheep, culled at 392 days of age, was confirmed PrP^d^ positive in brain. This sheep was the offspring of a non-infected F1 born to an orally dosed Romney sheep that developed BSE (Figure 1).

### Sheep of susceptible *PRNP* genotypes exposed to natural maternal and horizontal transmission

These animals included the F1 (*n* = 78) of orally dosed sheep that developed BSE and the F2 (*n* = 1) of infected F1. Fourteen of these 79 sheep (17.7%) showed evidence of BSE infection when examined at post-mortem. The following is an analysis of these transmissions within the different breed/genotype groups.

Of the 23 M_112_M ARQ Suffolk sheep that developed BSE after oral infection, 11 did not lamb, in most cases because they succumbed to BSE prior to first lambing (only one was a male). Of the remaining 12, seven gave birth to 11 F1 progeny that did not develop BSE and the other five had seven F1, five of which became infected (Table 2). Similarly, only 33 (32 orally dosed and one F1) of the 54 BSE-infected ARQ Romney ewes (47 orally dosed and seven F1) lambed and of those, only six dams raised seven infected progeny out of eight born to them. Overall, a total of 45 BSE-infected ARQ sheep had susceptible progeny (*n* = 61) but only 11 of them (24.4%) reared BSE infected offspring. However, 12 of 15 sheep (80%) born to these 11 dams succumbed to BSE. Of the 6 BSE-infected VRQ Cheviot sheep, two did not lamb, two had BSE-negative progeny (*n* = 10) and the other two produced seven F1 lambs, two of which were shown to be BSE infected at post-mortem examination. The ARs in the progeny of infected dams was not statistically different between ARQ Suffolk (5/18, 27.8%), ARQ Romney (7/43, 16.3%) and VRQ Cheviot (2/17, 11.8%) sheep. Of the 28 BSE-infected AHQ Cheviot sheep, eleven were males and only one of the 17 ewes lambed; the only lamb born to that ewe did not show evidence of BSE by 1276 days of age.

**Table 2:** Details of lambing and attack rates in progeny.

Genotype and breed	BSE +ve sheep that lambed	BSE +ve dams with +ve offspring (A)	Attack rates in offspring	
			Born to (A)	Total born
ARQ Suffolk	12/23 (52.2%)	5/12 (41.7%)	5/7 (71.4%)	5/18 (27.8%)
ARQ Romney^a^	33/54 (61.1%)	6/33 (18.2%)	7/8 (87.5%)	7/43 (16.3%)
VRQ Cheviot	4/6 (33%)	2/4 (50.0%)	2/7 (28.6%)	2/17 (11.8%)
AHQ Cheviot	1/28 (3.6%)	0/1 (0.0%)		0/1 (0%)
Total	50/111 (45.0%)	13/50 (26.0%)	14/22 (63.6%)	14/79 (17.7%)

^a^Includes the 47 sheep developing BSE after oral infection and the seven BSE-infected F1, one of which had a negative descendant (Figure 1). Only 50 BSE infected sheep had progeny that stayed in the flock for sufficient time; the other 61 were either males, died to BSE before lambing, or lambed sheep that died at too young an age to be considered (see text). Of the 50 ewes that lambed only 13 (A) had progeny of which some sheep (14/22) went on to develop BSE; the remaining 37 ewes did not produce any positive offspring amongst 57 sheep born to them

Overall, 50 BSE infected ewes lambed in a 7 year period (2003–2009). Eighty-eight lambs were born to those dams, of which nine did not survive for long enough to be considered (see “Materials an methods”), 14 went on to develop BSE and 65 did not show evidence of infection. The number of lambs born to BSE infected ewes in the different years was: 2003, 13; 2004, 5; 2005, 23; 2006, 19; 2007, 14; 2008, 12; 2009, 2.

### Statistical analysis of the efficiency of the different transmissions

The AR for *PRNP* susceptible sheep orally dosed with cattle BSE (104/113, 92.0%) was substantially higher than that in the offspring (F1 + F2) of infected sheep, whether all dams are considered (14/79, 17.7%, odds ratio [OR] 53.6, 95% confidence interval [CI] 21.9-131.1, *P* < 0.001) or only those raising BSE infected progeny are taken into account (14/22, 63.3%, OR 6.6, 95% CI 2.2–19.9, *P* < 0.01).

Despite differences in ARs between experimental oral challenge and natural maternal transmission, the mean STs of the BSE-affected ARQ Suffolk F1 (787 ± 101 days) and ARQ Romney F1 sheep (870 ± 154 days) were not significantly longer than those observed in the parental stock (Table 1; Figure 2). A similar comparison could not be made for VRQ Cheviot F1, since only one of them died of clinical BSE at 1804 days (the other infected offspring was detected when culled at 1883 days in the absence of clinical signs). However, the STs of naturally infected ARQ Suffolk progeny (787 ± 101 days) were significantly longer (*P* < 0.01) than those of the ARQ Suffolk lambs dosed with 1 g of cattle BSE shortly after birth (575 ± 87 days; Table 1; Figure 2).

The risk of developing BSE was more than 35 times higher (relative risk (RR) 36.3, 95% CI 4.8–271.8, *P* < 0.001) for sheep born to infected ewes (14/79, 17.7%) compared to animals born to uninfected ewes (1/205, 0.5%, including the 58 sheep of the parental stock; Table 1).

### Factors affecting the likelihood of maternal transmission of BSE

**Figure 3:**
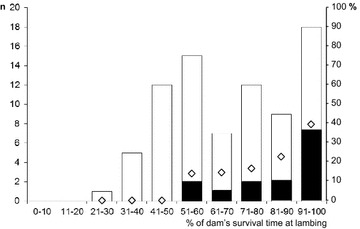
**Outcome of BSE infection in the offspring as a factor the proximity of lambing time to death of the dam.** Black and white bars represent the number of lambs (Y1 axis) that did or did not develop BSE, respectively. Diamonds represent the same data as percentage (Y2 axis). Note that BSE infected progeny were born only to dams that lambed at 50% or later of their survival time and that the proportion of offspring that went on to develop BSE (efficient maternal transmission) increased with the proximity between parturition and death of the dam due to BSE.

Within the progeny of infected ewes, the likelihood of infection appeared to be influenced by the degree of proximity between lambing and the death of the dam due to BSE (Figure 3). No BSE positive lambs arose from 18 births occurring in the first 50% of ST of orally dosed dams, seven infected lambs were bred from 43 (16.3%) born in the 51–90% range of dam ST and 38.9% (7/18) of lambs born in the last 10% of dam’s ST became infected. In absolute terms, the ARs in the F1 offspring were highest amongst lambs born within less than 100 days from the death of the ewe (9/22, 40.9%), intermediate in those born between 100 and 300 days of the dam’s death (3/15, 20%) and lowest for those born earlier than 300 days before the dam’s death (2/42, 4.8%).

Fisher’s test analysis of those figures indicated a significantly higher probability of progeny becoming infected for dams lambing in the last 10% of their survival period (RR 3.4, 95% CI 1.4–8.4, *P* < 0.05) compared with the rest (7/61, 11.5%). Similarly, the risk of developing BSE was significantly higher for lambs born within 100 days of the dam’s death (RR 4.7, 95% CI 1.8–12.4, *P* < 0.01) than for the rest (5/57, 8.8%).

Two further analyses were carried out to try to explain why some of the offspring born from dams that were close to their death went on to develop BSE whereas some other did not. These analyses were focused on the 16 dams that lambed within 100 days of their death due to BSE and produced 9 infected and 13 non infected offspring (Table 3). In the first analysis it was found that the time that elapsed between lambing and death of the dam was not significantly different between the eight that produced 9/12 infected offspring (55 ± 28.4 days) and the other eight raising 0/10 infected progeny (60 ± 26.2 days). The second analysis was driven by the notion that the spread and severity of PrP^d^ accumulation in LRS tissues can be taken as surrogate for the degree of systemic prion infection. In this analysis it was found that, at post-mortem examination of the dams, the sum of PrP^d^ scores in six LRS tissues was significantly higher (*P* < 0.05) in the first group of dams (10.8 ± 1.8) than in the second (7.3 ± 3.5).

**Table 3:** Details of dams that died of BSE within 100 days of final lambing.

ID	Offspring	Time intervals			Lymphoreticular system involvement						
		Dpi AL	ST	Diff.	RphLN	PTon	Spleen	MsnLN	PrsLN	DIle	LRS
S1	P	621	664	43	2	2.5	2	2	1.5	2.5	12.5
S2	P and N	623	677	54	3	2	1.5	1	0.5	2.5	10.5
S3	P and N	623	664	41	2.5	2	2	1.5	2	2	12.0
S5	P	496	570	74	2	2	2	2	2	2	12.0
R2	P	709	800	91	0.5	1.5	2	0.5	0.5	2.5	7.5
R5	P	703	706	3	2	3	2	0.5	0.5	2	10.0
R7	P and P	862	911	49	2.5	3	2	1	1.5	2.5	12.5
C1	P and N	1941	2026	85	3	1.5	1	0	1.5	2	9.0
Total	9/12	Mean ± SD		55 ± 28.4					Mean ± SD		10.8 ± 1.8
S4	N and N	983	1034	51	0.2	0.5	1.5	0	0.2	0.2	2.6
R1	N	706	786	80	2	2.5	2	0.5	0.5	2	9.5
R3	N	704	755	51	2	2.5	2	0	0.5	2.5	9.5
R4	N	705	766	61	0.5	3	2	1	2	3	11.5
R6	N	704	756	52	2	2	2.5	0.5	1	1.5	9.0
R8	N	861	871	10	1.5	1.5	1	1	1	1.5	7.5
R9	N	658	730	72	0.5	1	3	0.5	0.2	2	7.2
C2	N and N	1564	1663	99	0	0	1.5	0	0	0	1.5
Total	0/10	Mean ± SD		60 ± 26.2					Mean ± SD		7.4 ± 3.5

SD, standard deviation; ID, sheep identification; S, ARQ suffolk; R, ARQ romney; C, VRQ Cheviot; P, BSE positive; N, BSE negative; Dpi AL, days post-oral challenge at lambing; ST, survival time; Diff., days difference between ST and Dpi AL; RphLN, medial retropharyngeal lymph node; PTon, palatine tonsil; MsnLN, mesenteric LN; PrsLN, prescapular LN; DIle, distal ileum (Peyer’s patches); LRS, sum of lymphoreticular system tissue scores

### Detection of PrP^d^ in placentas and biopsies

**Figure 4:**
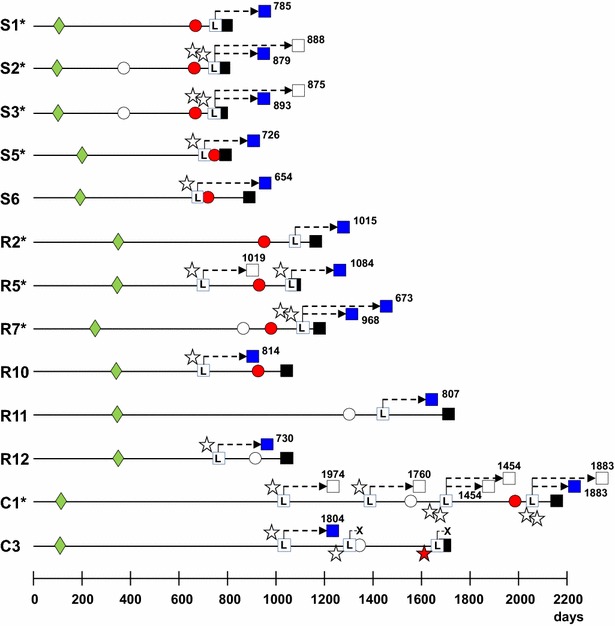
**Diagrammatic representation of the progression of BSE infection in 13 dams that produced the 14 infected offspring.** (S, ARQ Suffolk sheep, R, ARQ Romney sheep, C, VRQ Cheviot sheep). *These dam ID numbers coincide with those in Table 3. Green diamonds, age at inoculation. Circles, biopsy result (white, last negative; red, first positive). L within white square, age at lambing. Stars next to lambing, placenta result (white, negative; red, positive). X, stillbirth. Black squares, dam’s age at death of BSE (note that some squares appear partially hidden because of proximity between lambing and death). Blue and white squares, age at death of infected and non-infected progeny, respectively (indicated by digits, discontinuous lines are not representative).

Placentas were available from 40 orally dosed ewes that went on to develop clinical disease and multiple fixed tissue samples were examined by IHC for PrP^d^ detection. From a total of 66 such placentas, PrP^d^ was not detected in any of the 45 placentas collected from 31 infected dams whose progeny did not develop BSE, despite the fact that many of those placentas were obtained after the dam had shown peripheral PrP^d^ accumulation in tonsil and/or RAMALT biopsies (results not shown). The other 21 placentas were obtained from 10 dams whose progeny (all or some) went on to develop BSE (Figure 4). Only one placenta showed PrP^d^ accumulation by IHC, despite the fact that 11/21 corresponded to lambs that developed BSE and were obtained either after (6/11) or close (2/11) to a positive tonsil/RAMALT biopsy result in the dam (Figure 4). The only PrP^d^-positive placenta was from a VRQ Cheviot lamb stillborn from a dam (C3 in Figure 4) that in two previous deliveries produced an animal that developed BSE and a stillbirth, both with negative placentas. All three biopsies examined from this dam were negative; the latest was taken 1 year after the birth of the lamb that became BSE infected and 1 year before the delivery of the positive placenta.

The birth of eight of the 14 lambs that developed BSE was preceded by a positive biopsy result in the dam (S1, S2, S3, R2, R5, R7 and C1 in Figure 4). In three cases (S5, S6 and R10), the positive biopsy was obtained after the birth of the BSE-positive lambs but there were no biopsies examined beforehand. One BSE positive lamb was born after a negative biopsy in the dam taken 125 days before lambing (R11) and two BSE positive lambs (R12 and C3) were born before a biopsy from the dam was examined with negative result. Biopsies were also taken from 34 BSE-infected dams that produced fully BSE-negative offspring, with 17 of those dams giving consistently negative biopsy results on a total of 36 biopsy specimens. The other 17 dams yielded positive biopsy result after examining a total of 28 biopsies (biopsies were only repeated until a positive result was obtained and ceased after that). Only two of those 17 dams gave a positive biopsy result before (around 4 months) lambing, another two did so within a month of lambing and the other 13 were biopsy positive well after (around 7–12 months) the lamb was born.

### Accumulation of PrP^d^ in BSE-infected dams and progeny

**Figure 5:**
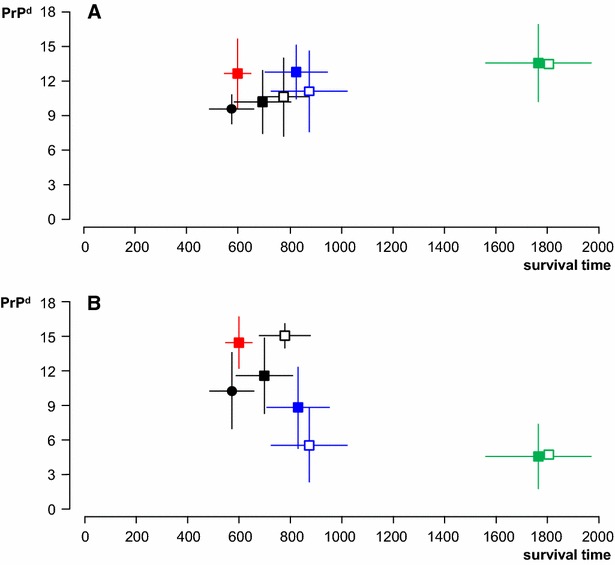
**Accumulation of PrP** ^**d**^ **in CNS and LRS tissues in relation to survival times.** Magnitude of PrP^d^ accumulation in the brain (**A**; Y axis as mean ± SD of individual sheep scores for total PrP^d^ (=sum of scores in seven standard brain areas)) and in LRS tissues (**B**; Y axis as mean ± SD of individual sheep scores for total PrP^d^ (=sum of scores in six different lymphoid tissues)) of sheep clinically affected with BSE after oral (solid symbols) or natural (open symbols) transmission in relation to their survival times (X axis in days, mean ± SD). Black, ARQ Suffolk sheep (circle, 1 g dose; square, 5 g dose); blue, ARQ Romney sheep; green, VRQ Cheviot sheep; red, AHQ Cheviot sheep. For number of animals in each group refer to Table 1. Note that (1) there is significant individual variation in PrP^d^ scores in both brain and LRS tissues, (2) brain PrP^d^ scores are relatively similar between groups regardless of differences in survival time and (3) LRS scores are low for VRQ Cheviot sheep despite their long survival time and high brain PrP^d^.

Terminal BSE cases showed extensive vacuolation of brainstem and PrP^d^ accumulation throughout the neuraxis and cerebellum with generally lower PrP^d^ levels in the cerebral cortex. However, the total magnitude of PrP^d^ accumulation in the brain differed markedly between individual sheep within a same breed/genotype/route of exposure group, making differences between groups not significant (Figure 5A). Moreover, the total magnitude of PrP^d^ accumulation in the brain did not appear to correlate with the survival time of the animals. Thus, the highest PrP^d^ levels were found in orally dosed VRQ Cheviot (13.5 ± 3.4), AHQ Cheviot (12.7 ± 3.0) and ARQ Romney (12.8 ± 2.3) sheep, which showed significant differences in survival times (see previous text and Figure 5A). Similarly, the magnitude of PrP^d^ deposition in the brain was similar for sheep orally dosed and their progeny, as exemplified by the PrP^d^ scores in ARQ Suffolk (10.7 ± 2.8 and 11.2 ± 3.4, respectively) and Romney (12.8 ± 2.3 and 11.2 ± 3.5, respectively) sheep.

In most clinical cases of BSE, significant PrP^d^ levels were also found in a range of LRS tissues. This is consistent with a generalised distribution of infectivity throughout the carcase by the time animals reached clinical end point. However, as in the brain, the abundance of PrP^d^ in LRS tissues also varied markedly between individuals and between groups (Figure 5B). It was also noted that the amount of PrP^d^ in the brain was unrelated to that in the LRS, so that the lowest LRS involvement was detected in orally dosed VRQ Cheviot sheep with the highest brain PrP^d^ (4.6 ± 2.8 and 13.5 ± 3.4, respectively), while naturally infected Suffolk sheep, with an intermediate PrP^d^ brain score, showed the highest magnitude of LRS PrP^d^ accumulation (11.2 ± 3.4 and 15.0 ± 1.1, respectively).

When performing discriminatory IHC with N- and C-terminal antibodies it was observed that the characteristic BSE truncation pattern (lack of reactivity of intracellular PrP^d^ with P4 antibody) was common to all sheep regardless of their breed, genotype and type of exposure. Equally, the BSE-characteristic PrP^d^ profile in brain (high levels of intraneuronal, intraglial, stellate and linear PrP^d^ types) was common to all sheep regardless of differing experimental factors.

## Discussion

This study provides compelling evidence that sheep BSE is naturally transmissible and contagious. This is in contrast to the report by Foster et al. [28], who did not find evidence of maternal transmission in nine susceptible offspring from four BSE-infected dams. In the present study, 14 lambs born to BSE infected dams and another born to a non-infected dam either developed clinical BSE or were positive for PrP^d^ when culled. Moreover, since the relative risk of infection was much higher (around 35 times) for the progeny of infected dams (~18%) than for the offspring of non-infected ewes (~0.5%), we conclude that, within the context of the experimental design of this study, natural BSE transmission occurs mostly from dam to offspring, that is, through a maternal or vertical route. The importance of maternal transmission as a means of maintaining endemic infection has previously been well documented for classical scrapie [20, 29–31], but there is neither epidemiological nor experimental evidence for maternal transmission of cattle BSE [32, 33].

It has recently been suggested that sheep foetuses may be infected with classical scrapie in utero [12–14]. In the present experiment, the lack of PrP^d^ detection in the placentas of the offspring that went on to develop BSE (Figure 4) and the fact that in some twin births only one lamb became infected (Table 3), do not suggest that maternal transmission occurred in utero. However, the relatively small SDs in the ST of the infected Suffolk and Romney F1 progeny, which were comparable to that in the corresponding orally dosed parental stock (Table 1; Figure 2), suggest that all those offspring became infected with similar infectious doses and at a similar time. Since STs of the offspring did not differ from that of the parental stock (Table 1; Figure 2), it is likely that infection occurred shortly after birth, and certainly before 3 months of age when lambs were separated from their dams. The precise maternal source of infectious agent for the progeny cannot be determined in this study. By comparison with classical scrapie, blood and other birth fluids, saliva [34] and milk [18, 19] are all potential sources of BSE agent. Infectivity in the last two secretions most probably reflects presence of the infectious agent in blood (prionaemia) rather than true replication of the agent in the salivary and mammary glands. This possibility is further suggested by the higher levels of PrP^d^ found in the LRS of dams with infected progeny compared to those with uninfected offspring (Table 3) and by the increased probability of infection in lambs born to ewes that lambed close to their death from BSE (Figure 3), that is, in advanced stages of the incubation period. This late preclinical disease stage is when dissemination of the infectious agent throughout the LRS is at its peak, as shown by studies on sheep BSE [7, 8, 10] and on sheep scrapie [35–38] and when prionaemia reaches highest levels, as demonstrated by blood transfusion experiments [39].

Lambs that were naturally infected from their dams, survived significantly longer than lambs receiving 1 g oral dose at 6–10 days of age (Figure 2). On one hand, this could be due to natural infection occurring at an older age than 10 days but still before weaning. On the other hand, it could suggest that the infection passed from dams to lambs was less that the equivalent titre represented by a 1 g oral infectious dose. This relatively low maternal infectious dose would also be in agreement with the significantly lower AR observed in the offspring compared to the orally dosed dams and with the fact that in some twin parturitions one lamb became infected and the other did not (Figure 4). However, a low infectious maternal dose may be difficult to reconcile with the fact that the survival times of the naturally infected progeny were actually no longer than those observed in their dams, which were given a 5 g dose. The explanation may lie in the age difference at infection between naturally infected offspring (pre-weaning) and their dams (average 5 months for Suffolk and 10 months for Romney sheep), which would make young lambs significantly more susceptible to BSE than weaned lambs and young adults, as has already been shown in a different study [9].

The results of this study thus show that BSE infected dams can transmit infection to their progeny. However, the excretion of the infectious agent is either discontinuous and/or its level variable. Thus, only some lambs –particularly those born at late preclinical stage of the dams—would receive an efficient dose and develop disease with relatively short incubation periods, while others would remain uninfected (at least as shown by PrP^d^ detection within the context of the study). Husbandry factors may also be important to explain the lack of transmission to some of the uninfected offspring. Dams incubating BSE were often poor mothers and, as is common practice in commercial farming, poorly mothered newborn lambs were often reared by other ewes, including sentinel controls and ewes at early BSE incubation stages. Efforts to trace back information about each individual lamb were unsuccessful because of the large scale of the study and its duration.

One sheep that was the progeny of a non-infected Romney F1 ewe was found to be BSE-infected. Assuming that there were no confounding management factors (e.g. that this lamb suckled from an infected ewe), this finding is indicative of the occurrence of horizontal or lateral BSE transmission. However, despite the flock being kept indoors for the duration of the study, the level of true horizontal transmission found in this study was very low (~0.5%) and certainly much lower than that observed for classical scrapie, where a high proportion of sheep of susceptible genotypes born to non-infected dams can become infected in a heavily contaminated environment [20]. A possible explanation for the difference between scrapie and sheep BSE in this respect lies in placental infectivity. In classical scrapie, a high proportion of placentas from infected ewes harbouring foetuses of susceptible genotype are infectious and/or contain detectable PrP^d^/PrP^res^ [16, 17, 40–43] and such infected placentas are regarded as one of the most important, if not the main, sources of environmental contamination and horizontal transmission [20, 44]. In contrast, in this study, where BSE infected dams had foetuses and placentas of the same susceptible genotypes, only one placenta was found to be PrP^d^ positive. This was despite many placentas corresponding to lambs that went on to develop BSE or others being collected once the dam had shown peripheral prion replication as demonstrated by PrP^d^ positive biopsy. Arrival of infectivity in the placenta is most likely through the haematogenous route and, since a high number of BSE infected dams showed LRS involvement around gestation, their placentas are likely to have been exposed to the infectious agent. This would therefore suggest that there is a difference between the scrapie and BSE agents’ ability to replicate in the placenta, although without proper comparative kinetics data between scrapie and BSE infected sheep such a possibility cannot be ascertained. Nevertheless, regardless of the pathogenetic mechanism, the almost complete lack of placental PrP^d^ detected in this study is probably a significant factor in the low rate of horizontal transmission observed.

In addition to placentas, environmental contamination at lambing time may be due to birth excreta such as blood and amniotic fluid from infected dams, which were not tested in the course of this study. Even if those fluids were infectious, the level of environmental contamination would have been low, since only a few (50 in 7 years) BSE infected dams lambed each year and even fewer (between 0 and 4 per year for a total of 13 in 7 years) lambed offspring that went on to develop BSE. In other words, pressure of infection at lambing time, which has been recognised as an important factor for sheep scrapie [20], would have been low in the experimental set up of this study. This would have contributed to low environmental contamination and negligible horizontal transmission, as observed.

In order to achieve endemic BSE within a flock, horizontal and/or maternal transmission of infection must be efficient. This was not the case in the present study. Only a single lamb became infected by putative horizontal transmission. Of 99 BSE orally infected ewes, 49 succumbed to BSE before lambing or had progeny that died at young age (without having lambed) and 50 lambed viable offspring. Thirty-seven of these dams, most of which lambed only once before succumbing to BSE, had an uninfected progeny. Of the remaining 13, one (C1 in Figure 4) had multiple parturitions with only one of six lambs becoming infected, one (R5) had two parturitions with only one of two lambs becoming infected and the remaining 11 ewes had only one lambing, with 12/14 lambs developing BSE. Only one of these ewes (R7) produced a set of twins both of which developed infection; the rest only reared single infected progeny. Moreover, of the 14 F1 progeny that developed BSE, only 6 sheep (2 each of ARQ Suffolk, ARQ Romney and VRQ Cheviot sheep) were females. In other words, out of almost a hundred infected ewes of the parental stock only slightly more than 6% of the sheep of the F1 generation were in a situation of transmitting infection to the next generation and the only one that lambed did not transmit. Thus, although the results of this study clearly indicate that natural transmission of BSE can occur, they do not provide strong evidence for multigenerational maintenance of infection. This conclusion would agree with the absence of any BSE-like case in a retrospective study on more than 2000 natural ovine TSEs diagnosed between 1998 and 2004 [45]. However, a perhaps important caveat of the present experiment is the rapidity with which orally dosed dams succumbed to BSE. In a different scenario, perhaps with a lower oral dose, infected dams could have had the opportunity to lamb in several successive seasons and this could have led to a higher opportunity to raise infected progeny and/or to increase environmental contamination at lambing time. Therefore, the possibility that ovine BSE may show greater trans-generational spread of infectivity under conditions of high infectivity pressure, which was not the case in this experiment, cannot be ruled out.

This study has also provided some additional information regarding the pathogenesis of sheep BSE after oral infection. Firstly, the resistance of M_112_T ARQ Suffolk sheep to oral BSE, which confirms previous reports [10, 22]; in this respect BSE differs from oral scrapie, for which the threonine polymorphism at codon 112 confers only partial resistance, both in terms of AR and ST [23]. Secondly, the poor correlation between clinical disease, magnitude of PrP^d^ in the brain and survival time (Figure 5A); this is in agreement with previous reports on experiments done by the oral [10] and the intracerebral [25] routes. Thirdly, that non-PrP genetics may be involved in the relative proportion of PrP^d^ in the brain and LRS tissues. Thus, when pooling data from parental stock and F1 together, ARQ Romney sheep showed higher brain PrP^d^ for lower LRS PrP^d^ compared with Suffolk sheep of the same genotype, which showed the reverse pattern (results not shown but can be inferred from Figures 5A and B); this is in agreement with the findings of other sheep BSE experiments [10, González et al., unpublished observations). Fourthly, that VRQ sheep appear to be, in terms of survival time, less susceptible to cattle BSE than to some forms of classical sheep scrapie, which might be related to low replication of the agent in the LRS (Figure 5B) of BSE- compared to scrapie-infected sheep (for review see [46]); however, long incubation periods in VRQ sheep have also been reported for sheep scrapie, when the infectious source is of a heterologous ARQ genotype [23]. Finally, that both in terms of truncation site of intracellular PrP^d^ and of brain PrP^d^ profile, BSE in the naturally infected animals is indistinguishable from that in the donor ewes; this finding is in agreement with those obtained on serial experimental passage of BSE in sheep [47].

In conclusion, the results of the present study show that transmission of BSE from dam to offspring may occur. However, the low efficiency of maternal transmission and the almost complete lack of horizontal transmission do not suggest that BSE infectivity is likely to be self-sustaining within sheep flocks, at least within the context of the experimental design reported here and the caveats already expressed.

## References

[1] Wilesmith WJ, Wells GAH, Cranwell MP, Ryan JB (1988). Bovine spongiform encephalopathy: epidemiological studies. Vet Rec.

[2] Wilesmith WJ, Ryan JB, Atkinson MJ (1991). Bovine spongiform encephalopathy: epidemiological studies on the origin. Vet Rec.

[3] Espinosa JC, Herva ME, Andréoletti O, Padilla D, Lacroux C, Cassard H, Lantier F, Castilla J, Torres JM (2009). Transgenic mice expressing porcine prion protein resistant to classical scrapie but susceptible to sheep bovine spongiform encephalopathy and atypical scrapie. Emerg Infect Dis.

[4] Eloit M, Adjou K, Coulpier M, Fontaine JJ, Hamel R, Lilin T, Messiaen S, Andréoletti O, Baron T, Bencsik A, Biacabe AG, Beringue V, Laude H, Le Dur A, Vilotte JL, Comoy E, Deslys JP, Grassi J, Simon S, Lantier F, Sarradin P (2005). BSE agent signatures in a goat. Vet Rec.

[5] Jeffrey M, Martin S, González L, Foster JD, Langeveld JPM, van Zijderveld FG, Grassi J, Hunter N (2006). Immunohistochemical features of PrP^d^ accumulation in natural and experimental goat transmissible spongiform encephalopathies. J Comp Pathol.

[6] Foster JD, Hope J, Fraser H (1993). Transmission of bovine spongiform encephalopathy to sheep and goats. Vet Rec.

[7] Jeffrey M, Ryder S, Martin S, Hawkins SAC, Terry L, Berthelin-Baker C, Bellworthy SJ (2001). Oral inoculation of sheep with the agent of bovine spongiform encephalopathy (BSE). 1. Onset and distribution of disease-specific PrP accumulation in brain and viscera. J Comp Pathol.

[8] van Keulen LJM, Vromans MEW, Dolstra CH, Bossers A, van Zijderveld FG (2008). Pathogenesis of bovine spongiform encephalopathy in sheep. Arch Virol.

[9] Hunter N, Houston F, Foster J, Goldmann W, Drummond D, Parnham D, Kennedy I, Green A, Stewart P, Chong A (2012). Susceptibility of young sheep to oral infection with bovine spongiform encephalopathy decreases significantly after weaning. J Virol.

[10] McGovern G, Martin S, Jeffrey M, Bellworthy SJ, Spiropoulos J, Green R, Lockey R, Vickery CM, Thurston L, Dexter G, Hawkins SAC, González L (2015). Influence of breed and genotype on the onset and distribution of infectivity and disease associated prion protein in sheep following oral infection with the bovine spongiform encephalopathy agent. J Comp Pathol.

[11] Low JC, Chambers J, McKelvey WA, McKendrick IJ, Jeffrey M (2009). Failure to transmit scrapie infection by transferring preimplantation embryos from naturally infected donor sheep. Theriogenology.

[12] Garza MC, Fernández-Borges N, Bolea R, Badiola JJ, Castilla J, Monleón E (2011). Detection of PrPres in genetically susceptible fetuses from sheep with natural scrapie. PLoS One.

[13] Foster JD, Goldmann W, Hunter N (2013). Evidence in sheep for pre-natal transmission of scrapie to lambs from infected mothers. PLoS One.

[14] Spiropoulos J, Hawkins SAC, Simmons MM, Bellworthy SJ (2014). Evidence of in utero transmission of classical scrapie in sheep. J Virol.

[15] Race R, Jenny A, Sutton D (1998). Scrapie infectivity and proteinase K-resistant prion protein in sheep placenta, brain, spleen, and lymph node: implications for transmission and antemortem diagnosis. J Infect Dis.

[16] Jeffrey M, Martin S, Thomson JR, Dingwall WS, Begara-McGorum I, González L (2001). Onset and distribution of tissue PrP accumulation in scrapie-affected suffolk sheep as demonstrated by sequential necropsies and tonsillar biopsies. J Comp Pathol.

[17] Andréoletti O, Lacroux C, Chabert A, Monnereau L, Tabouret G, Lantier F, Berthon P, Eychenne F, Lafond-Benestad S, Elsen JM, Schelcher F (2002). PrP(Sc) accumulation in placentas of ewes exposed to natural scrapie: influence of foetal PrP genotype and effect on ewe-to-lamb transmission. J Gen Virol.

[18] Konold T, Moore SJ, Bellworthy SJ, Simmons HA (2008). Evidence of scrapie transmission via milk. BMC Vet Res.

[19] Konold T, Moore SJ, Bellworthy SJ, Terry LA, Thorne L, Ramsay A, Salguero FJ, Simmons MM, Simmons HA (2013). Evidence of effective scrapie transmission via colostrum and milk in sheep. BMC Vet Res.

[20] González L, Dagleish MP, Martin S, Finlayson J, Sisó S, Eaton SL, Witz J, Hamilton S, Pang Y, Steele P, Reid HW, Chianini F, Jeffrey M (2012). Factors influencing temporal variation of scrapie incidence within a closed Suffolk sheep flock. J Gen Virol.

[21] Bellworthy SJ, Dexter G, Stack M, Chaplin M, Hawkins SAC, Simmons MM, Jeffrey M, Martin S, González L, Hill P (2005). Natural transmission of BSE between sheep within an experimental flock. Vet Rec.

[22] Saunders GC, Lantier I, Cawthraw S, Cawthraw S, Berthon P, Moore SJ, Arnold ME, Windl O, Simmons MM, Andréoletti O, Bellworthy S, Lantier F (2009). Protective effect of the T112 PrP variant in sheep challenged with bovine spongiform encephalopathy. J Gen Virol.

[23] González L, Jeffrey M, Dagleish MP, Goldmann W, Sisó S, Eaton SL, Martin S, Finlayson J, Stewart P, Steele P, Pang Y, Hamilton S, Reid HW, Chianini F (2012). Susceptibility to scrapie and disease phenotype in sheep: cross-Prnp genotype experimental transmissions with natural sources. Vet Res.

[24] González L, Dagleish MP, Martin S, Dexter G, Steele P, Finlayson J, Jeffrey M (2008). Diagnosis of preclinical sheep scrapie in live sheep by the immunohistochemical examination of rectal biopsies. Vet Rec.

[25] González L, Martin S, Houston FE, Hunter N, Reid HW, Bellworthy SJ, Jeffrey M (2005). Phenotype of disease-associated PrP accumulation in the brain of bovine spongiform encephalopathy experimentally infected sheep. J Gen Virol.

[26] Thuring CMA, Erkens JHF, Jacobs JG, Bossers A, van Keulen LJM, Garssen GJ, van Zijderveld FG, Ryder SJ, Groschup MH, Sweeney T, Langeveld JP (2004). Discrimination between scrapie and bovine spongiform encephalopathy in sheep by molecular size, immunoreactivity, and glycoprofile of prion protein. J Clin Microbiol.

[27] Martin S, González L, Chong A, Houston FE, Hunter N, Jeffrey M (2005). Immunohistochemical characteristics of disease-associated PrP are not altered by host genotype or route of inoculation following infection of sheep with bovine spongiform encephalopathy. J Gen Virol.

[28] Foster JD, Goldmann W, McKenzie C, Smith A, Parnham DW, Hunter N (2004). Maternal transmission studies of BSE in sheep. J Gen Virol.

[29] Hoinville LJ (1996). A review of the epidemiology of scrapie in sheep. Rev Sci Tech.

[30] Elsen JM, Amigues Y, Schelcher F, Ducrocok V, Andréoletti O, Eychenne F, Tien Khang JV, Poivey JP, Lantier F, Laplanche JL (1999). Genetic susceptibility and transmission factors in scrapie: detailed analysis of an epidemic in a closed flock of Romanov. Arch Virol.

[31] Hoinville LJ, Tongue SC, Wilesmith JW (2010). Evidence for maternal transmission of scrapie in naturally affected flocks. Prev Vet Med.

[32] Wilesmith WJ, Wells GAH, Ryan JBM, Gavier-Widen D, Simmons MM (1997). A cohort study to examine maternally-associated risk factors for bovine spongiform encephalopathy. Vet Rec.

[33] Wrathall AE, Brown KFD, Sayers AR, Wells GAH, Simmons MM, Farrelly SSJ, Bellerby P, Squirrell J, Spencer YI, Wells M, Stack MJ, Bastiman B, Pullar D, Scatcherd J, Heasman L, Parker J, Hannam DAR, Helliwell DW, Chree A, Fraser H (2002). Studies of embryo transfer from cattle clinically affected by bovine spongiform encephalopathy (BSE). Vet Rec.

[34] Maddison BC, Rees HC, Baker CA, Taema M, Bellworthy SJ, Thorne L, Terry LA, Gough KC (2010). Prions are secreted into the oral cavity in sheep with preclinical scrapie. J Infect Dis.

[35] van Keulen LJM, Schreuder BEC, Vromans MEW, Langeveld JPM, Smits MA (2000). Pathogenesis of natural scrapie in sheep. Arch Virol Suppl.

[36] Tabouret G, Lacroux C, Lugan S, Costes P, Corbière F, Weisbecker JL, Schelcher F, Andréoletti O (2010). Relevance of oral experimental challenge with classical scrapie in sheep. J Gen Virol.

[37] González L, Pitarch JL, Martin S, Thurston L, Moore J, Acín C, Jeffrey M (2014). Identical pathogenesis and neuropathological phenotype of scrapie in valine, alanine, glutamine/valine, alanine, glutamine sheep infected experimentally by oral and conjunctival routes. J Comp Pathol.

[38] González L, Pitarch JL, Martin S, Thurston L, Simmons H, Acín C, Jeffrey M (2014). Influence of polymorphisms in the prion protein gene on the pathogenesis and neuropathological phenotype of sheep scrapie after oral infection. J Comp Pathol.

[39] Houston F, McCutcheon S, Goldmann W, Chong A, Foster J, Sisó S, González L, Jeffrey M, Hunter N (2008). Prion diseases are efficiently transmitted by blood transfusion in sheep. Blood.

[40] Pattison IH, Hoare MN, Jebbett JN, Watson WA (1972). Spread of scrapie to sheep and goats by oral dosing with foetal membranes from scrapie infected sheep. Vet Rec.

[41] Lacroux C, Corbière F, Tabouret G, Lugan S, Costes P, Mathey J, Delmas JM, Weisbecker JL, Foucras G, Cassard H, Elsen JM, Schelcher F, Andréoletti O (2007). Dynamics and genetics of PrP^sc^ placental accumulation in sheep. J Gen Virol.

[42] Hamilton S, Finlayson J, Pang Y, Buxton D, Eaton S, Steele P, Dagleish M, Benavides J, González L, Jeffrey M, Sisó S, Reid H, Chianini F (2008) PrPsc distribution in perfused placentas from Suffolk sheep naturally infected with scrapie. In: Proceedings of the Prion Conference 2008, Madrid, October 2008, pp 82

[43] Garza-García MC (2012) Aportaciones al conocimiento de la patogenia y transmisión del scrapie en infección natural. PhD Thesis, University of Zaragoza

[44] Healy AM, Hannon D, Morgan KL, Weavers E, Collins JD, Doherty ML (2004). A paired case-control study of risk factors for scrapie in Irish sheep flocks. Prev Vet Med.

[45] Stack MJ, Jeffrey M, Gubbins S, Grimmer S, González L, Martin S, Chaplin M, Webb P, Simmons M, Spencer Y, Bellerby P, Hope J, Wilesmith J, Matthews D (2006). Monitoring for bovine spongiform encephalopathy in sheep in Great Britain, 1998–2004. J Gen Virol.

[46] Jeffrey M, González L (2007). Classical sheep transmissible spongiform encephalopathies: pathogenesis, pathological phenotypes and clinical disease. Neuropathol Appl Neurobiol.

[47] Stack M, González L, Jeffrey M, Martin S, Macaldowie C, Chaplin M, Thorne J, Sayers R, Davis L, Bramwell J, Grimmer S, Bellworthy S (2009). Three serial passages of BSE in sheep do not significantly affect discriminatory test results. J Gen Virol.

